# Vitamin D and Ovarian Cancer: Systematic Review of the Literature with a Focus on Molecular Mechanisms

**DOI:** 10.3390/cells9020335

**Published:** 2020-02-01

**Authors:** Andraž Dovnik, Nina Fokter Dovnik

**Affiliations:** 1 University Clinic for Gynaecology and Obstetrics, University Medical Centre Maribor, Ljubljanska 5, 2000 Maribor, Slovenia; 2 Department of Oncology, University Medical Centre Maribor, Ljubljanska 5, 2000 Maribor, Slovenia; nfokter@gmail.com

**Keywords:** vitamin D, ovarian cancer, vitamin D receptor, vitamin D response elements

## Abstract

Vitamin D is a lipid soluble vitamin involved primarily in calcium metabolism. Epidemiologic evidence indicates that lower circulating vitamin D levels are associated with a higher risk of ovarian cancer and that vitamin D supplementation is associated with decreased cancer mortality. A vast amount of research exists on the possible molecular mechanisms through which vitamin D affects cancer cell proliferation, cancer progression, angiogenesis, and inflammation. We conducted a systematic review of the literature on the effects of vitamin D on ovarian cancer cell.

## 1. Introduction

Vitamin D is a lipid soluble vitamin involved in calcium and phosphate homeostasis [1]. By promoting the intestinal absorption of calcium and bone osteoclast activity it helps to keep calcium and phosphate concentrations within physiological range [1]. In recent decades, a vast amount of research has been performed on potential extraskeletal roles of vitamin D. Available data indicate that there might be an association between vitamin D levels and the risk of developing different kinds of cancers [2]. Epidemiologic evidence suggests that lower circulating vitamin D levels are associated with a higher risk of ovarian cancer [3]. Furthermore, a recent meta-analysis showed that vitamin D supplementation is associated with a 13% decrease in cancer mortality [4].

**Figure 1:**
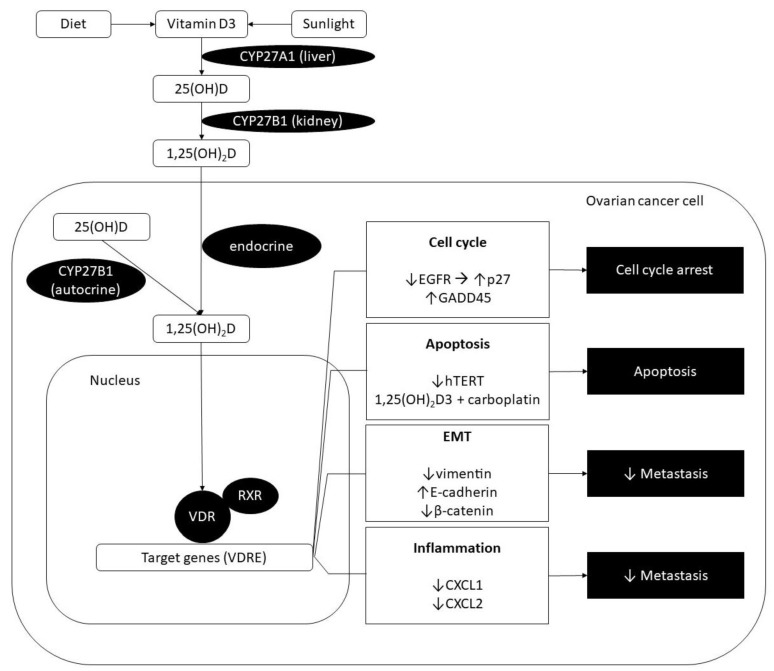
Schematic representation of the effects of vitamin D on the ovarian cancer cell. The two main sources of vitamin D are sunlight and dietary products. Vitamin D3 is transported to the liver where it is hydroxylated to 25-hydroxyvitamin D (25(OH)D) with the microsomal and mitochondrial 25-hydroxylase encoded by the gene CYP27A1. 25(OH)D is further metabolised in the kidneys with the action of 1α-hydroxylase encoded by the gene CYP27B1, forming the active metabolite 1,25-dihydroxyvitamin D (1,25(OH)_2_D) (calcitriol). 1,25(OH)_2_D can also be formed in the mitochondria of the ovarian cancer cell. The physiological effects of 1,25(OH)_2_D are carried out through interaction with the vitamin D receptor (VDR). This is a nuclear transcription factor and when activated with 1,25(OH)_2_D undergoes hetero-dimerisation with a retinoic acid X receptor (RXR). Then, this complex binds to the specific DNA sequences known as vitamin D response elements (VDRE). The interaction with VDRE results in downregulation or upregulation of genes involved in regulation of the cell cycle, apoptosis, epithelial–mesenchymal transition (EMT), immune responses, and inflammation. Only the pathways that have been studied specifically in ovarian cancer are represented. EGFR: Epidermal growth factor receptor; hTERT: Human telomerase reverse transcriptase.

The two main sources of vitamin D are sunlight and dietary products (Figure 1) [1]. Under the influence of sunlight, previtamin D3 is formed in the skin and then undergoes a heat induced isomerisation to vitamin D3 [1]. Vitamin D3 is then transported to the liver attached to vitamin D binding protein (DBP) [5]. There, it is hydroxylated to 25-hydroxyvitamin D (25(OH)D) with the microsomal and mitochondrial 25-hydroxylase encoded by the gene CYP27A1 [1,6,7]. 25(OH)D is the circulating form of vitamin D and its concentration is measured when determining vitamin D status. 25(OH)D is further metabolised in the kidneys with the action of 1α-hydroxylase encoded by the gene CYP27B1, forming the active metabolite 1,25-dihydroxyvitamin D (1,25(OH)_2_D) (calcitriol) [6,8]. The production of calcitriol also takes place in the bone, placenta, dendritic cells, parathyroid gland, prostate, and cancer cells [7,9,10].

The physiological effects of 1,25(OH)_2_D are carried out through interaction with the vitamin D receptor (VDR) (Figure 1) [1]. This is a nuclear transcription factor and when activated with 1,25(OH)_2_D undergoes hetero-dimerisation with a retinoic acid X receptor (RXR) [1,11,12,13]. Then, this complex binds to the specific DNA sequences known as vitamin D response elements (VDRE) [1,11]. The interaction with VDRE results in downregulation or upregulation of genes which are involved in apoptosis, cell growth and differentiation, immune responses, and inflammation (Figure 1) [1,12]. The activity of 1α-hydroxylase is regulated by respective concentrations of 1,25(OH)_2_D: With increasing concentrations of this active metabolite the enzymatic activity together with the expression of CYP27B1 decreases [6]. The rate limiting step for degradation of 25(OH)D and 1,25(OH)_2_D is the activity of the catabolic enzyme 24-hydroxylase encoded by the gene CYP24A1 which transforms these two molecules to inactive forms which are soluble in water and can therefore be excreted in the bile [1,6].

The main role of calcitriol is maintaining calcium homeostasis. VDR is located in the small intestine and when the VDR-RXR complex is formed it interacts with VDRE in the genes associated with intestinal calcium absorption. If intestinal absorption is inadequate to maintain calcium homeostasis vitamin D mobilises skeletal calcium by binding with VDR in osteoblasts. 1,25(OH)_2_D increases the expression of RANK ligand (RANKL) in osteoblasts. RANKL then binds with RANK on proosteoclasts and induces their transformation to mature osteoclasts [2].

Ovarian cancer is a heterogenous group of diseases. The most common is epithelial ovarian cancer which represents 90% of all cases [14]. This is an aggressive form of gynaecological malignancy with the 5-year overall survival rate of less than 50% [3,14]. The most common histological subtype is serous adenocarcinoma and about 80% of serous cancers are diagnosed in advanced stages [3,14]. The risk of developing ovarian cancer is decreased by the use of oral contraceptives, higher parity, tubal sterilisation and removal of the ovaries, and increased by the use of hormones during menopause [14]. 

We performed a systematic review of the literature on molecular mechanisms through which vitamin D can influence ovarian cancer cells. The literature search was conducted using the MEDLINE electronic database for the search terms (“vitamin d” OR “ergocalciferols”) AND (“ovarian neoplasms” OR “ovarian cancer”) and identified 251 papers published until October 2019. Peer-reviewed articles published in the English language and containing an abstract were considered and reference lists were screened for additional relevant citations. All articles dealing with molecular mechanisms were included in the review. Full-text versions of all manuscripts were obtained. The systematic review was conducted in accordance to PRISMA guidelines.

## 2. Epidemiological Data on the Link between Vitamin D and Ovarian Cancer

The role of vitamin D in the development and progression of cancer has been the subject of many studies. Higher vitamin D concentrations have been associated with a lower risk of developing different kinds of cancers including ovarian cancer [7,15]. However, the epidemiological evidence linking vitamin D concentrations and ovarian cancer incidence and/or mortality is less strong than the evidence concerning some other cancer types [16]. In 2010, a systematic review of the literature reported that approximately half of the ecologic and case-control studies found reductions in ovarian cancer incidence or mortality with increasing geographic latitude, solar radiation levels, or vitamin D supplementation, while cohort studies found no overall risk reduction with increasing consumption or plasma levels of vitamin D prior to diagnosis [17]. A meta-analysis of four cohort studies reported a possible inverse association of ovarian cancer incidence and circulating 25(OH)D levels that did not reach statistical significance [18]. Mixed results were obtained in more recent ecologic studies [19,20], case-control [21], and cohort studies [22,23] as well. A Mendelian randomization study found that European women with genetically lowered 25(OH)D concentrations due to single nucleotide polymorphisms (SNPs) had higher susceptibility for ovarian cancer [3].

## 3. Overview of the Role of Vitamin D and VDR in Ovarian Cancer

In vitro studies have established the role of vitamin D in inhibiting the proliferation of cancer cells [7,24,25]. Calcitriol regulates tumour growth by arresting various stages of the cell cycle [25,26]. Ovarian cancer cells can be arrested in the G1/S checkpoint by the action of calcitriol on cyclin-dependent kinase (CDK) inhibitors P21 and P27 [26,27]. 1,25(OH)_2_D can also induce cell cycle arrest at the G2/M checkpoint acting primarily on GADD45 [24,26]. In addition, calcitriol seems to have a role in epithelial–mesenchymal transition [28]. It has been shown to increase the expression of the epithelial marker E-cadherin and reduce the expression of EMT transcription factors. The net effect of these actions was inhibition of migration and invasion of SKOV-3 cells [28]. Furthermore, calcitriol has been associated with the mechanisms promoting cell apoptosis by downregulation of telomerase in ovarian cancer cells [29]. Vitamin D has also been shown to play a role in anti-inflammatory processes in ovarian cancer. Since inflammation is a risk factor in carcinogenesis, this can also be viewed as one of its anti-tumour activities [26]. Moreover, it has been reported that vitamin D has an effect on fatty acid and glucose metabolism in cancer cells [30,31]. 

The vitamin D receptor is present in normal ovarian epithelium and ovarian cancer cells [12]. The expression of VDR has been found to be increased in ovarian cancer tissue compared to normal ovarian tissue [32]. VDR in the ovary is involved in the regulation of the aromatase gene expression and oestrogen biosynthesis. Higher levels of gonadotropins, reduced expression of the aromatase gene and a lower aromatase activity were detected in VDR-null mice [12]. 

VDR polymorphisms and the associated ovarian cancer risk have been extensively investigated in the past [12,33,34,35,36,37,38,39]. The most studied variants include FokI, BsmI, Cdx-2, and TaqI [15].

The following chapters will discuss in detail the molecular mechanisms through which vitamin D could influence the occurrence of ovarian cancer. Studies on the effects of vitamin D specifically on ovarian cancer cells are summarised in Table 1.

## 4. Cell cycle and Programmed Cell Death (Apoptosis)

Calcitriol as the active form of vitamin D has been shown to have an effect on cancer growth by regulating different stages of the cell cycle [24,25,27]. The regulation of cell cycle progression occurs through checkpoints by the help of cyclin-dependent kinases (CDK) which are activated by cyclins and deactivated by CDK inhibitors [50].

Progression of the cell cycle through the G1/S checkpoint is regulated along the CDK2-Rb-E2F axis. Cyclin dependent kinase transcription is stimulated by the epidermal growth factor (EGF) which leads to the activation of E2F, degradation of p27, and progression from G1 to the S phase [25,27]. Calcitriol has been found to regulate the expression of p27, cyclin A, cyclin E, and Skp2 in human ovarian cancer cell lines [25]. The main target for calcitriol is p27, a tumour suppressor whose accumulation in the cell leads to cell cycle arrest in the G1 phase. The DNA synthesis in the S phase of the cell cycle is controlled by cyclin A and other E2F regulated genes. Calcitriol decreases the expression of cyclin A resulting in the decrease of CDK2 activity and decreased phosphorylation of p27. This decreases the affinity of p27 for Skp2 ubiquitin ligase which causes the accumulation of p27 in the cell leading to cell cycle arrest [25].

In addition to the effect of vitamin D on the proteins of the cell cycle signaling pathway, epidermal growth factor receptor (EGFR) has been identified in human ovarian cancer cell lines as a target gene leading to a sequential reaction which stops the cell cycle at the G1/S checkpoint [27]. A novel VDRE has been found in intron 1 of the EGFR genome. It has been shown that 1,25(OH)_2_D suppresses EGFR expression at mRNA and protein levels. The downregulation of EGFR caused by calcitriol leads to a decreased response of ovarian cancer cells to the epidermal growth factor [27]. Another important gene identified in ovarian cancer cell lines found to be involved in the tumour suppressor activity of calcitriol is GADD45 [24]. This is a p53 regulated gene and has an important role in DNA repair and the cell cycle [24]. When calcitriol binds to its receptor, the VDR-RXR complex leads to the accumulation of GADD45 in the cell causing a decrease in the Cdc2 activity and preventing progression to the M phase of the cell cycle [24].

In summary, it has been shown that 1,25(OH)_2_D decreases the growth of multiple ovarian cancer cell lines. However, the concern has been raised that therapeutic concentrations of 1,25(OH)_2_D would cause hypercalcaemia in humans [41]. American researchers have reported on the antiproliferative activity of the 1,25(OH)_2_D analogue EB1089. They have shown that this is even more effective than 1,25(OH)_2_D in regulating tumour growth and inducing apoptosis by increasing the transcription of GADD45 in ovarian cancer cell lines. Importantly, EB1089 suppressed tumour growth in nude mice models without inducing hypercalcaemia [41].

The effect of 1,25(OH)_2_D on ovarian cancer cell apoptosis has also been reported [29]. 1,25(OH)_2_D has been shown to downregulate telomerase, the enzyme required to stabilise telomere length and enable cell immortality, thereby inducing apoptosis in ovarian cancer cell lines [29]. High telomerase activity in cancer cells is primarily due to overexpression of its catalytic subunit, human telomerase reverse transcriptase (hTERT), which is increased up to 100-fold in cancer compared to non-cancer cells. 1,25(OH)_2_D decreases the level of hTERT mRNA by decreasing its stability [29]. Another vitamin D target regulating telomerase function is microRNA-498 [42,51]. MicroRNA are single stranded RNA molecules which usually lower gene expression. A VDRE has been found in the 5′ region of the microRNA-498 genome. When activated by 1,25(OH)_2_D, miR-498 lowers the mRNA expression of hTERT by binding to its 3′-untranslated region, thus leading to apoptosis [42].

On the other hand, 1,25(OH)_2_D has been found to regulate the extrinsic apoptotic pathway through inhibition of the tumour necrosis factor-related apoptosis-inducing ligand (TRAIL) and TRAIL receptor 4 and downregulation of the Fas ligand [40]. Zhang et al. have demonstrated that pretreatment with 1,25(OH)_2_D decreases apoptosis induced by Fas ligand and TRAIL [40]. The authors have emphasised the need to understand the potential adverse effects of vitamin D on ovarian cancer cells. Subsequent analysis within the same study has shown that this adverse effect could be overcome by the molecular manipulation of death receptors TRAIL 4 and Fas [40].

Moreover, a synergistic effect between 1,25(OH)_2_D and carboplatin has been reported, showing that 1,25(OH)_2_D enhances apoptosis induced by carboplatin [44]. The effect of 1,25(OH)_2_D on apoptosis has also been shown in microarray studies on ovarian cancer cell lines [52]. The pro-apoptotic effect of 1,25(OH)_2_D was carried out through decreased expression of apoptosis inhibitor 5 (API5L1) and increased expression of pro-apoptotic c-abl oncogene 1, transforming growth factor β (TGF-β), and receptor tyrosine kinase ABL1 [52].

## 5. Epithelial–Mesenchymal Transition and Cancer Progression

Epithelial–mesenchymal transition (EMT) is a reversible process where epithelial cells gain mesenchymal morphology and lose intercellular contacts, becoming more invasive and able to migrate. This has been postulated to play a critical role in ovarian cancer progression [28]. This phenomenon has been studied on SKOV-3 cells stimulated by the TGF-β1. This cytokine promotes tumour progression in advanced stages by various mechanisms including EMT. 1,25(OH)_2_D decreased the expression of the mesenchymal marker vimentin and increased the expression of the epithelial marker E-cadherin. It also caused an inhibition of SKOV-3 cell migration and inhibited TGF-β1 induced EMT [28]. In addition, in vivo and in vitro studies have suggested that 1,25(OH)_2_D and VDR help inhibit the spread of ovarian cancer to the omentum [45]. A study on mouse ovarian surface epithelial cells found that 1,25(OH)_2_D delayed their malignant transformation by decreasing the expression of β-catenin and increasing the expression of E-cadherin [48].

The vitamin D binding protein has also been reported to have a role in ovarian cancer progression, namely in cancer invasiveness, formation of ascites, and cancer metastasis [5]. Animal studies have shown that exposure of ovarian cancer cells to vitamin D3 before the inoculation to immunodeficient mice decreased the potential of the cells to metastasise into liver, lung, and bone marrow [47].

DDX4 (DEAD (Asp-Glu-Ala-Asp)-box helicase 4) has been found to be another target for active vitamin D [49]. Treatment with active vitamin D reduced the expression of DDX which inhibited the invasion of ovarian cancer cells [49]. Microarray studies have identified a number of target genes involved in tumour growth and progression regulated by 1,25(OH)_2_D. The upregulation of lysophosphatidic acid G-protein coupled receptor (Edg2) is consistent with its function in the inhibition of tumour growth. On the other hand, calcitriol downregulates growth-promoting chemochines IL-8, GRO-β, and GRO-γ [52].

## 6. Angiogenesis

We have not found any studies specifically evaluating the effect of vitamin D on angiogenesis in ovarian cancer. Nevertheless, in human cancer cells, 1,25(OH)_2_D has been found to have an antiangiogenic effect by modulating the hypoxia-inducible factor 1 (HIF-1) pathway [53]. Hypoxia is the main trigger of angiogenesis in tumours and HIF-1 is the key transcription factor regulating angiogenesis. It has been shown that 1,25(OH)_2_D reduces the expression of the HIF-1α subunit and of the vascular endothelial growth factor and inhibits cancer cell proliferation under hypoxic conditions [53]. Other research has shown that the antiangiogenic effect of 1,25(OH)_2_D on tumour endothelial cells is also VDR mediated [54]. Increased vascular volume and a lower number of pericytes which regulate the proliferation of endothelial cells were found in VDR knock-out animals [54].

## 7. Immunomodulation and Tumour Inflammatory Response

Vitamin D is an important regulator of the innate immune system that acts by inducing several antimicrobial peptides, influencing chemotaxis, autophagy, and phagolysosomal fusion of monocytes and macrophages, increasing the physical barrier function of epithelial cells and influencing the composition of gut microbiota [55]. In addition, vitamin D modulates the adaptive immune system, mostly acting as an immunosuppressor by downregulating T helper (Th) 1 cells, inhibiting the production of several proinflammatory cytokines, upregulating Th2 cells and T regulatory (Treg) cells, downregulating Th17 cells, and modulating antigen-presenting dendritic cells into a “tolerogenic state” [55]. Low vitamin D levels have thus been associated with an increased susceptibility for infections, as well as an increased risk for several autoimmune diseases [55].

The relationship between cancer and the immune system is complex, ranging from tumoricidal effector response of immune cells to immune tolerance and prometastatic effects [56].

Some cancers are infiltrated by the cells of the adaptive and innate immune system [57]. These immune cells can have a major effect on cancer progression by supplying growth factors, factors which contribute to angiogenesis, EMT, and survival factors which help escape apoptosis [57]. The inflammatory response is important in the development of ovarian and other types of cancers [46,58]. Cyclooxygenase 1 and 2 (COX-1 and COX-2) are two isoenzymes which mediate the synthesis of prostaglandin and therefore control the inflammatory response [46,59]. While COX-1 is expressed constitutively, the expression of COX-2 is regulated by prostaglandins, growth factors, and cytokines. The upregulation of COX-2 in the development of ovarian cancer is associated with decreased apoptosis, increased cell proliferation, and neoangiogenesis [59]. Increased expression of COX-2 in ovarian cancer cells compared to benign tissue has been reported and has been associated with reduced overall survival [60,61]. A study on ovarian and endometrial cancer cell lines has shown that calcitriol and progesterone decreased the expression of pro-inflammatory cytokines CXCL1 and CXCL2 leading to downregulation of proteins associated with the process of metastasis [43]. CXCL1 and CXCL2 are expressed in a greater degree in ovarian cancer and have been found to be associated with metastasis, angiogenesis, and tumour growth in breast and squamous cell cancers [43]. The anti-proliferative effects of COX-2 inhibitors have been established in both in vitro and in vivo studies. Furthermore, it has been shown that calcitriol and the COX-2 inhibitor celecoxib have synergistic effects on ovarian cancer cell proliferation [46].

On the other hand, pre-existing antitumour T-cell response is associated with a greater efficacy of immune checkpoint blockade, whereas the so-called immune deserted tumours hardly ever respond to immunotherapy [62]. It has recently been shown that tumour intrinsic Wnt/β-catenin signaling mediates immune exclusion in melanoma and this has been confirmed across other human cancers [63,64]. Vitamin D-VDR signaling inhibits the Wnt/β-catenin pathway and could be explored therapeutically with the aim of counteracting resistance to immunotherapy [63,64].

## 8. Enzyme Expression and Tumour Metabolism

The enzymes participating in vitamin D metabolism 25-hydroxylase, 1α-hydroxylase, and 24-hydroxylase have been shown to be upregulated in ovarian cancer cells in one study [32], but other studies have failed to confirm this finding [65,66]. In one of the latter, decreased expression of CYP27B1, the gene encoding for 1α-hydroxylase, in primary and metastatic ovarian cancer has been associated with decreased overall survival [66]. The exposure of ovarian cancer cell lines to 1,25(OH)_2_D has shown no effect on the expression of 25-hydroxylase and 1α-hydroxylase but has resulted in the upregulation of 24-hydroxylase mRNA [67]. In studies on ovarian cancer cell lines, high concentrations of 1,25(OH)_2_D inhibited the growth of these cells while lower concentrations stimulated their growth [68]. It has been postulated that higher concentrations were required due to the activity of 24-hydroxylase which inactivates 1,25(OH)_2_D. The growth stimulating effect of lower calcitriol concentrations may be due to the effect of metabolites produced by 24-hydroxylase, although their role is still poorly understood [68].

We have not found any research about the direct effect of 1,25(OH)_2_D specifically on ovarian cancer cell metabolism. The effect of 1,25(OH)_2_D on the metabolism of other cancer types has been reported [26].

## 9. Vitamin D receptor

Ovarian tumours and normal ovarian epithelium both express VDR [7,12] but its expression is increased in ovarian cancer tissue compared to normal ovarian tissue [32]. Moreover, VDR levels on platelets have been found to be higher in patients with ovarian tumours compared to healthy women and higher in women with ovarian malignancies compared to benign ovarian tumours [69]. As mentioned above, VDR is involved in the biosynthesis of oestrogen and control of the expression of the aromatase gene [7]. Studies on ovarian cancer cell lines have shown that 1,25(OH)_2_D upregulates the expression of VDR and has an inhibitory effect on the growth of ovarian cancer cells [70]. Furthermore, cooperation between VDR and the androgen receptor, both of which regulate ovarian cancer cell growth, has been demonstrated. Specifically, 1,25(OH)_2_D upregulated the androgen receptor and dihydrotestosterone inhibited the growth of cancer cells by upregulation of VDR [70].

Vitamin D receptor polymorphisms have been studied in various populations with controversial results [12,33,34,35,36,37,38,39]. The most studied single nucleotide polymorphisms (SNP) were FokI, BsmI, Cdx-2, Apa1, and TaqI [12,15].

FokI is located on exon 2. The presence of F allele on FokI leads to a three amino acid longer protein compared to the protein produced by the F allele [71,72]. The longer protein has been reported to be less responsive to 1,25(OH)_2_D with a lower transcription activity [12,71]. Carriers of the F allele have been shown to have a two times higher risk of developing ovarian cancer compared to homozygous carriers of the F allele. However, this association was present only in Caucasian and not in Japanese women [12]. A subsequent meta-analysis showed that the F allele is most common in the Asian population, followed by the Caucasian and African populations [72]. Moreover, homozygous FF allele ovarian cancer patients have been found to have better overall survival compared to Ff and ff allele carriers [34]. The association between FokI and ovarian cancer risk has been confirmed in some [35,36,37,38], but not all subsequent analyses [33,37,39,73]. Nevertheless, recent meta-analyses all indicate that the FokI polymorphism is associated with increased ovarian cancer risk [15,71,72,74,75,76,77].

The other most commonly studied VDR polymorphism is BsmI [7]. It is located on intron 8 and its polymorphisms have been associated with skin and colorectal cancers [71]. Some research has suggested a role of the BsmI receptor polymorphisms in the development of ovarian cancer [39,73] but not all studies support this finding [12,33,35,37]. Meta-analyses have provided controversial results [15,71,74,76,77,78,79]. Most of them found no association between BsmI and ovarian cancer [15,71,74,76]. However, the most recent meta-analysis indicated that the association was present, especially in the Caucasian population [77].

In one study, the Cdx-2 polymorphism was associated with an increased risk of developing ovarian cancer in Japanese women but not in other ethnic groups [12]. This association has not been confirmed by other studies [35,37]. A meta-analysis evaluating the role of the Cdx-2 polymorphism in the development of different types of cancer indicated that the Cdx-2 polymorphism was not associated with increased cancer risk in the Asian and Caucasian populations but was associated with increased cancer risk in the African-American population [80]. Specifically, Cdx-2 polymorphisms have been associated with increased risk of developing ovarian and colorectal cancer [80]. Two other meta-analyses found increased ovarian cancer risk in heterozygous and dominant models for Cdx-2 polymorphisms [76,77] while a third meta-analysis did not find any association [15].

The ApaI polymorphism has also been investigated and case control studies have provided mixed results [12,33,37,81]. A study on 170 cases of epithelial ovarian cancer compared to healthy controls found no association between the ApaI polymorphism and ovarian cancer risk [33]. In another case control study, the ApaI polymorphism was associated with increased risk of ovarian cancer in African-American women [81]. Other researchers reported that the ApaI polymorphism seems to be associated with ovarian cancer in women with higher predicted 25(OH)D levels but not in women with lower predicted levels [82]. A meta-analysis showed the ApaI polymorphism to be associated with ovarian cancer risk in the European population [74], but other meta-analyses found no such association [15,71,76,77].

The majority of case control studies have not found any association between the TaqI polymorphism and ovarian cancer risk [12,33,37]. One study reported on higher ovarian cancer risk with the TaqI polymorphism only in women with higher predicted 25(OH)D levels [82]. Meta-analyses have shown no such association [15,71,74,76,77].

Further research is needed to confirm the role of vitamin D polymorphisms in the risk of ovarian cancer. However, the accumulating evidence of the association of FokI and ovarian cancer makes VDR a potential target for cancer prevention [15].

## 10. Use of Calcitriol and its Analogues in Inhibition of Ovarian Cancer Cell Growth

As previously mentioned, Zhang et al. reported that the vitamin D analogue EB1089 inhibited the growth of ovarian cancer cells via its effect on GADD45 [41]. Brard et al. showed a cytotoxic effect of the non-hypercalcaemic vitamin D analogue MT19c on ovarian cancer cell lines [83]. This derivative did not achieve cytotoxicity through its effect on VDR but through its effect on the insulin-like growth factor receptor (IGFR) and the tumour necrosis factor alpha (TNFα) receptor on ovarian cancer cells [83]. The effect of MT19c was subsequently analysed in vivo by the same authors [84]. Human ovarian cancer cell lines were inoculated subcutaneously in mice. After treatment with MT19c the majority of mice responded completely and achieved prolonged tumour free survival [84]. Another non-hypercalcaemic vitamin D analogue studied in vitro and in vivo was PT19c, showing inhibition of growth of ovarian cancer xenografts in mice without inducing hypercalcaemia [85]. Furthermore, the calcidiol derivative B3CD showed anti-proliferative and cytotoxic effects on SKOV-3 cell lines in vitro [86]. Additionally, in vivo studies in nude mice demonstrated complete responses in 25% and tumour regression in the majority of animals [86]. Moreover, the addition of calcitriol to Müllerian inhibiting substance (MIS) in vitro potentiated the effect of MIS on ovarian cancer cell proliferation [87]. The studies on the use of calcitriol and its analogues for the inhibition of ovarian cancer cell growth are summarised in Table 2.

## 11. Cancer Stem Cells

The effect of vitamin D on ovarian cancer stem cells has also been studied [88,89]. Emerging evidence indicates the role of stem cells in ovarian cancer metastasis formation, tumour relapse, and development of chemoresistance [88]. Vitamin D has been shown to reduce the self-renewing capacity of cancer stem cells. Active vitamin D increased the expression of VDR and cytoplasmic β-catenin and reduced the expression of stemness-associated gene CD44 in vitro [88]. In addition, calcitriol has been reported to decrease the number of cancer stem cells via a VDR mediated pathway through inhibition of the Wnt pathway [89].

## 12. Conclusions

This review provides the latest insights into molecular mechanisms through which vitamin D could influence ovarian cancer on the cellular level. Epidemiologic evidence indicates that adequate vitamin D levels are associated with a lower risk of ovarian cancer and reduced cancer mortality in general. The systematic review of the literature has not identified any human studies regarding the effect of vitamin D or its analogues on ovarian cancer patients and such supplementation or treatment cannot be recommended for this indication. However, further research is warranted based on the encouraging in vitro and in vivo data. In the future, the clinical role of vitamin D supplementation in the prevention or treatment of cancer patients should be explored in randomized controlled trials.

## Figures and Tables

**Table 1:** Description of studies on the effects of calcitriol and its analogues specifically on ovarian cancer cell cycle, apoptosis, invasion, and inflammatory process. See text for details. NA: Not available.

First Author (Year of Publication)	Type of Study	Molecular Mechanism	Biologic Effect	Ref. No.
Jiang (2003)	In vitro	↑GADD45 → ↓Cdc2	G2/M cell cycle arrest	[24]
Jiang (2004)	In vitro	↓hTERT → ↓telomerase	↑Apoptosis	[29]
Li (2004)	In vitro	↓Cyclin E → ↓CDK2 → ↑p27	G1/S cell cycle arrest	[25]
Zhang (2005) (I)	In vitro	↓TRAIL, ↓TRAIL-R4, ↓Fas	↑Apoptosis	[40]
Zhang (2005) (II)	In vitro In vivo	↑GADD45	↑Apoptosis, G2/M cell cycle arrest	[41]
Shen (2011)	In vitro	↓EGFR → ↑p27	G1/S cell cycle arrest	[27]
Kasiappan (2012)	In vitro In vivo	↑microRNA-498 → ↓hTERT	↑Apoptosis	[42]
Kavandi (2012)	In vitro	↓CXCL1, ↓CXCL2	↓ Expression of proinflammatory cytokines → downregulation of proteins associated with metastasis	[43]
Zhang (2014)	In vitro	Possibly reactive oxygen species (ROS) production	↑Apoptosis (synergistic effect with carboplatin)	[44]
Lungchukiet (2015)	In vitro In vivo	NA	Suppress ovarian cancer invasion to omentum	[45]
Thill (2015)	In vitro	NA	Inhibition of cellular proliferation together with COX-2 inhibitor celecoxib	[46]
Abdelbaset-Ismail (2016)	In vitro In vivo	NA	↑Apoptosis, anti-proliferative effect, ↓migration	[47]
Hou (2016)	In vitro	↑E-cadherin, ↓Vimentin, ↓β-catenin	↓Metastatic potential	[28]
Liu (2016)	In vitro In vivo	↑E-cadherin, ↓β-catenin	Delay in progression (↓metastatic potential)	[48]
Chen (2018)	In vitro	↓DDX4	Anti-proliferative effect, ↓migration	[49]

**Table 2:** Description of studies on the use of calcitriol and its analogues for the inhibition of ovarian cancer cell growth. See text for details.

First Author (Year of Publication)	Type of Study	Analogue	Biological Effect	Ref. No.
Zhang (2005)	In vitro In vivo	EB1098	Inhibition of ovarian cancer cell growth without inducing hypercalcemia in vivo	[41]
Lange (2010)	In vitro In vivo	Calcidiol derivative B3CD	Anti-proliferative effect on ovarian cancer cell lines; tumour regression in majority of animals	[86]
Brard (2011)	In vitro	MT19c	Cell cycle arrest DNA fragmentation ↑Apoptosis	[83]
Moore (2012)	In vivo	MT19c	Prolonged tumour free survival	[84]
Kawar (2013)	In vitro In vivo	PT19c	Inhibition of growth of ovarian cancer xenografts in mice without inducing hypercalcaemia	[85]
Jung (2016)	In vitro	Calcitriol and Müllerian inhibiting substance (MIS)	Inhibition of ovarian cancer cell growth	[87]
